# Ultra-High Resolution 9.4T Brain MRI Segmentation via a Newly Engineered Multi-Scale Residual Nested U-Net with Gated Attention

**DOI:** 10.3390/bioengineering12101014

**Published:** 2025-09-24

**Authors:** Aryan Kalluvila, Jay B. Patel, Jason M. Johnson

**Affiliations:** 1 Weinberg College of Arts and Sciences, Northwestern University, Evanston, IL 60201, USA; 2 Athinoula A. Martinos Center for Biomedical Imaging, Department of Radiology, Massachusetts General Hospital, Boston, MA 02129, USA; jbpatel@alum.mit.edu; 3 Department of Radiology and Biomedical Imaging, Yale University, New Haven, CT 06520, USA; jason.johnson@yale.edu

**Keywords:** magnetic resonance imaging (MRI), GA-MS-UNet++ (Gated Attention, Multi-Scale Residual U-Net++), deep learning (DL), machine learning (ML)

## Abstract

A 9.4T brain MRI is the highest resolution MRI scanner in the public market. It offers submillimeter brain imaging with exceptional anatomical detail, making it one of the most powerful tools for detecting subtle structural changes associated with neurological conditions. Current segmentation models are optimized for lower-field MRI (1.5T–3T), and they struggle to perform well on 9.4T data. In this study, we present the GA-MS-UNet++, the world’s first deep learning-based model specifically designed for 9.4T brain MRI segmentation. Our model integrates multi-scale residual blocks, gated skip connections, and spatial channel attention mechanisms to improve both local and global feature extraction. The model was trained and evaluated on 12 patients in the UltraCortex 9.4T dataset and benchmarked against four leading segmentation models (Attention U-Net, Nested U-Net, VDSR, and R2UNet). The GA-MS-UNet++ achieved a state-of-the-art performance across both evaluation sets. When tested against manual, radiologist-reviewed ground truth masks, the model achieved a Dice score of 0.93. On a separate test set using SynthSeg-generated masks as the ground truth, the Dice score was 0.89. Across both evaluations, the model achieved an overall accuracy of 97.29%, precision of 90.02%, and recall of 94.00%. Statistical validation using the Wilcoxon signed-rank test (*p* < 1 × 10^−5^) and Kruskal–Wallis test (H = 26,281.98, *p* < 1 × 10^−5^) confirmed the significance of these results. Qualitative comparisons also showed a near-exact alignment with ground truth masks, particularly in areas such as the ventricles and gray–white matter interfaces. Volumetric validation further demonstrated a high correlation (R^2^ = 0.90) between the predicted and ground truth brain volumes. Despite the limited annotated data, the GA-MS-UNet++ maintained a strong performance and has the potential for clinical use. This algorithm represents the first publicly available segmentation model for 9.4T imaging, providing a powerful tool for high-resolution brain segmentation and driving progress in automated neuroimaging analysis.

## 1. Introduction

Magnetic resonance imaging is a critical tool for characterizing brain anatomy and pathology. It offers important insight into the structural and functional aspects of several neurological conditions [1]. Accurate segmentation of the brain structures in MRI helps physicians gain critical insights into pathological changes associated with severe neurological conditions. Historically, there have been two leading players in the MRI space: 1.5T and 3T [2]. Large public databases, such as the Human Connectome Project, focus solely on these two sequence types, leading researchers to concentrate on creating algorithms for just those two [3]. Recently, however, ultra-high-field MRI scanners, particularly those operating at 7T and above, have revolutionized the field of brain imaging. They offer significantly better signal-to-noise ratio (SNR) and intensity contrasts, as well as improved visualization of fine anatomical details and better detection of small abnormalities and lesions, such as those seen in multiple sclerosis [4,5]. Unfortunately, high-field scanners are prohibitively costly, and as such, very few hospitals in the United States have been able to deploy 9.4T MRI scanners [6]. These scanners, however, offer unprecedented spatial resolutions down to submillimeter levels (0.6–0.8 mm isotropic) and improve tissue contrast. Existing segmentation models struggle to cope with the 9.4T data because they were trained on lower resolution imaging [7].

### 1.1. Existing Deep Learning Frameworks

Current state-of-the-art deep learning models include the U-Net, Attention U-Net, Very Deep Super Resolution model (VDSR), R2UNet, and Nested U-Net architecture. The U-Net was the first breakthrough architecture in biomedical segmentation. Proposed by Ronneberger et al., this approach employed a symmetrical encoder and decoder structure with skip connections, preserving spatial information from previous layers [8]. Variations such as the 3D U-Net extend this basic architecture into three dimensions but struggle with GPU efficiency [9]. The Attention U-Net is a good example of how components of the original U-Net can be enhanced to better address specific tasks. Researchers dynamically weighted different regions of the image and focused their computational resources on contrast and anatomical structures [10]. This approach significantly improved the model’s ability to delineate subtle boundaries. The Very Deep Super Resolution (VDSR) model was proposed by Kim et al., and it introduced a deep convolutional architecture for single-image super resolution that uses residual learning to help improve training and accuracy. Its feature extraction power has inspired the use of it for segmentation purposes [11]. The R2UNet architecture, proposed by Alom et al., combines the strengths of both recurrent residual convolutional layers with the standard U-Net architecture [12]. Integrating residual units and recurrent connections is a powerful way the R2UNet improves feature representation. It also promotes strong segmentation performance, especially in the cases of complex biomedical images (such as 9.4T). The Nested U-Net, also known as UNet++, further extends the U-Net architecture by utilizing nested and dense skip connections between the encoder and decoder pathways, offering superior accuracy for biomedical segmentation tasks, particularly in brain MRI segmentation [13].

### 1.2. Volumetry in Brain MRI

Quantitative volumetric analysis aids in the early detection of neurodegenerative diseases. For instance, reduced hippocampal volume is a clear characteristic of Alzheimer’s disease and can be one of the first indications prior to cognitive decline [14]. Similarly, changes in brain volume can help differentiate between conditions such as Alzheimer’s disease, Parkinson’s disease, and another behavioral variant of frontotemporal dementia. In multiple sclerosis (MS) or other diseases involving white matter lesions, brain volume loss correlates with a substantial decline in cognitive ability and disability, making volumetric assessment crucial for evaluating treatment efficacy [15,16]. The integration of automated volumetric analysis tools, such as NeuroQuant, has further revolutionized clinical practice by providing rapid and reproducible measurements, reducing inter-rater variability, and enhancing diagnostic accuracy [17]. These advancements have made brain volumetry a standard component in the evaluation of patients with cognitive impairments. As mentioned previously, the ultra-high resolution provided by the 9.4T MRI enables the detection of subtle anatomical changes that are often undetectable at lower field strengths. This increased sensitivity enables more precise measurements of brain volumes, providing even greater specificity in detecting neurodegenerative disorders.

Similarly, volumetric analysis plays a pivotal role in the clinical workflow for tumor patients [18,19]. Automated segmentation pipelines using conventional MR such as T1 and T2 imaging have been used in a wide range of applications, including for primary, metastatic, and pediatric brain tumors [20,21,22,23,24]. More recently, work has been performed using less common MR modalities. For instance, a recent systematic review on computer-aided diagnosis using hyperspectral imaging for brain cancer reported a high diagnostic performance despite small datasets and limited external validation [25]. Finally, even though we focus on brain MRI in this manuscript, we note that automated deep learning-based pipelines have use in a diverse range of applications, including but not limited to cardiac MRI, abdominal CT, retinal fundus imaging, and chest X-rays [26,27,28,29].

### 1.3. Clinical Importance of 9.4T

The 9.4T MRI offers ultra-high resolution in brain imaging by capturing a submillimeter resolution [30]. This ultra-high resolution enables clinicians and researchers to identify subtle abnormalities that may have been missed by lower-strength scanners, such as 1.5T and 3T. The enhanced tissue contrast has been shown to improve the detection of small lesions, which is particularly important for the early diagnosis of conditions. Additionally, 9.4T provides stronger and more accurate mapping of white matter pathways, aiding in the understanding of brain connectivity and neurological disorders. The increased sensitivity also enables better functional imaging properties, providing clearer insights into brain activity. Although primarily used for research purposes currently, we hope that, by developing robust segmentation models for 9.4T data, its potential can be realized and further applied in clinical practice. As technology advances and accessibility improves, this tool is likely to become a key component of clinical imaging, helping to detect neurological conditions earlier and support more targeted treatments.

## 2. Materials and Methods

### 2.1. Study Design

This study utilizes MRI data that was collected from the UltraCortex dataset [30]. It utilized brain MRI scans at ultra-high resolution, obtained at 9.4T by the Max Planck Institute for Biological Cybernetics. A total of 78 subjects whose MRI scans were analyzed for cortical segmentation were included, comprising data from 78 healthy adult volunteers (28 females and 50 males, aged between 20 and 53 years). The demographic data were collected in accordance with the guidelines and ethical standards of the institution, and all participants provided written informed consent, which the relevant ethics committees of the University Hospital, Tübingen, Germany, approved. Specific subjects were excluded based on the following criteria: (1) known neurological or psychiatric diseases, (2) contraindications to MRI, (3) abnormal vision, and (4) severe motion artifacts or failed technical validation of images.

### 2.2. Image Acquisition

The MRI scans were obtained using a whole-body 9.4T MRI scanner (produced by Siemens Healthineers, Malvern, PA, USA) at the Department of High Field Magnetic Resonance in Malvern, PA. These scans employed T1-weighted MP-RAGE and MP2RAGE sequences with spatial resolutions ranging from 0.6 to 0.8 mm, and the total dataset comprised 86 T1-weighted images from 78 unique subjects. For each participant, multiple acquisitions were performed to account for motion artifacts and ensure the most precise sequence was obtained for analysis.

### 2.3. Dataset Preprocessing

MRI images were stored in NIfTI format, and each scan and respective mask were accessed using the nibabel library. Out of the 78 total subjects, only 12 subjects had associated manual segmentation masks that could be utilized for training and inference. The segmentation masks were binary and represented delineations between white and gray matter boundaries (not including cerebrospinal fluid). This subset was randomly split into eight subjects for training and four subjects for testing. The image and mask paths were stored, with each MRI scan being loaded slice by slice (in a 2D fashion) to promote efficient memory usage. The images were normalized by subtracting the mean and dividing by the standard deviation to ensure consistent scaling across the dataset.

To further improve the model’s robustness, data augmentation was applied during training. Random horizontal flips and rotations were applied to both the images and the masks to ensure that the model learned to generalize across various orientations. These augmentations were only applied to the training set, whereas the testing set remained unchanged to ensure valid evaluation. Each axial slice was resized to 256 × 256 pixels using bicubic interpolation. This resizing was applied in the image (pixel) space rather than the physical (millimeter) space. Given that the original voxel spacing ranged from 0.6 mm to 0.8 mm, the resulting voxel spacing after resizing is proportionally adjusted relative to the original dimensions of each image volume. They were converted into PyTorch tensors (version 2.7.1) and returned alongside their corresponding masks. This pipeline ensured that the MRI images and masks were processed correctly and augmented, providing high-quality data for training and testing the models.

While incorporating the complete 3D volume could potentially enhance anatomical consistency, GPU memory limitations restricted us from inputting entire volumes into the model. Furthermore, our goal was to develop a fast, easily deployable model tailored explicitly for 9.4T brain MRI. Therefore, we opted to work with 2D axial slices instead. Even though central axial slices of the brain have a more rich anatomical detail than peripheral slices, we did not find any benefit in using a slice sampling scheme weighted towards central slices. Thus, in our implementation, every available axial slice (including peripheral slices with little to no ground truth segmentation present) from each subject’s T1-weighted volume was used for training.

On the 74 patients which did not have manual segmentations, we utilize SynthSeg to generate automatic segmentations of the target region [31]. We note that SynthSeg only runs on 1 mm isotropic resolution data. Thus, we first downsample our ultra high-resolution images to 1 mm isotropic to use with SynthSeg, and subsequently upsample the output segmentations via nearest-neighbor interpolation back to the original imaging resolution, which is between 0.6 and 0.8 mm. These outputs served as a reference point for validating our model’s performance in the absence of extensive manual ground truth annotations. The SynthSeg-generated ground truth labels were also produced in the same format as the manual ground truth labels provided with the dataset, ensuring consistency in structure and compatibility. To further validate their suitability, all generated segmentations were subjected to a visual quality control step, during which we manually inspected the outputs to confirm anatomical plausibility and to ensure that no gross errors or artifacts were present.

### 2.4. Neural Network Engineering

**Figure 1:**
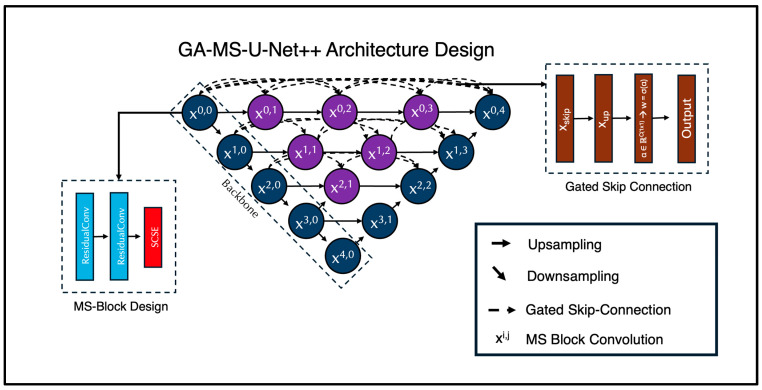
Proposed GA-MS-UNet++ Architecture with Multi-Scale-Block and Gated Skip Connection designs mapped out. Our novel architecture includes these components in order to bolster performance and improve efficiency of the model. We modified the original Nested U-Net architecture by injecting these components within the skip connections and the standard convolutions.

For the deep learning portion of this study, we expanded on the traditional Nested U-Net architecture. It integrates multiple scales for multi-feature learning and has demonstrated exceptional performance in brain MRI segmentation tasks, as it can handle both fine-grained and large-scale anatomical details. In this paper, we introduce the Gated Multi-Scale Nested U-Net (GA-MS-UNet++) (Figure 1). This is an improvement over the standard UNet++ with three key innovations: (1) multi-scale residual blocks, (2) attention mechanisms, and (3) gated skip connections. The architecture still follows the encoder–decoder structure, where the encoder path progressively downsamples the input using stacked Multi-Scale Blocks (MSBlocks).

Each MSBlock contains two Residual Convolution Units (RCUs) and one Spatial Channel Squeeze and Excitation (SCSE) attention mechanism. Each RCU is composed of two convolutional layers with kernel size 3. We modify the second convolutional layer to use a dilation value of 2, broadening the receptive field of the network without increasing the number of trainable parameters. This multi-scale context aggregation enables the network to capture additional fine details and global structure. We utilize group normalization (GN) instead of BatchNorm (to stabilize training on small batches) and Leaky ReLU activation functions. A residual connection is applied by adding the input to the output of the convolutional sequences. If the input and output channels differ, a 1 × 1 convolution is used to align the dimensions. Following the two RCU layers, a Spatial Channel Squeeze and Excitation (SCSE) attention mechanism is used to recalibrate feature maps. The channel attention part uses global average pooling to help condense each channel into a scalar, then passes this vector through 1 × 1 convolutions with a reduction ratio r = 4 and applies a sigmoid activation to produce channel-wise attention weights. Mathematically, it is represented as follows:

cSE(x)=x⋅σ(W2⋅ReLU(W1⋅GAP(x)))

Here, *GAP(X)* performs the global pooling average, and W1 and W2 are the learnable weights of the convolutions. The spatial attention then applies the 1 × 1 convolution to produce a single spatial attention map:

sSE(x)=x⋅σ(Ws ∗ x)

The final output of the SCSE block is the sum of the added tensors:

SCSE(x)=cSE(x)+sSE(x)

After each set of two MSBlocks per layer, downsampling in the encoder is handled via a 2 × 2 max pooling operation. The decoder is symmetrical to the encoder, using two MSBlocks per layer to reinforce multi-scale context for all stages of reconstruction. Upsampling in the decoder is handled via a bilinear interpolation operation.

We leveraged the nested skip design path from the regular Nested U-Net, which generates multiple intermediate decoder outputs. However, unlike the traditional UNet++, which concatenates skip features, the GA-MS-UNet++ utilizes gated skip connections to help learn how much information to retain from the encoder features versus the upsampled decoder features. Each gate has a learnable parameter, which is initialized to zero, and the skip connection is computed as follows:

GatedSkip(xskip,xup)=σ(α)⋅xskip+(1−σ(α))⋅Conv1x1(xup)

Here, σ(α) generates a per-channel attention mask that facilitates the blending of the encoder and decoder signals. The final segmentation map is produced by applying a 1 × 1 convolution to the final decoder output.

Each skip connection pathway had a learnable gate parameter that determined how much information to pass from the encoder feature map versus the upsampled decoder feature. These gates were initialized to 0.5, ensuring that, at the start of training, the contribution from the encoder and decoder pathways was balanced equally. The gate parameters were then optimized with the rest of the network using backpropagation, allowing the model to learn whether to favor encoder features, decoder features, or a mixture of both.

### 2.5. Training Parameters

A batch size of 1 was used due to memory constraints and the large size of the input images, with training performed for 100 epochs on a NVIDIA H100 GPU. The model was optimized using the Adam optimizer, with a learning rate of 1 × 10^−4^ and a weight decay of 1 × 10^−5^, minimizing binary cross-entropy loss between the predicted masks and the ground truth. The predictions were thresholded at 0.5 to generate the binary segmentation outputs. After training, the model’s performance was evaluated using the Dice coefficient, precision, accuracy, recall, and Structural Similarity Index Measure (SSIM). These metrics were computed from both the raw predictions and their binarized forms to assess both pixel-wise accuracy and perceptual quality.

As an additional validation step, we also compared all 12 manually annotated MRI scans against their corresponding SynthSeg-generated segmentations. This comparison produced an average Dice coefficient of 0.9480 with a standard deviation of 0.0050, which demonstrated a strong concordance between the automated and expert-derived labels.

## 3. Results

### 3.1. Dice and SSIM Evaluation

**Figure 2:**
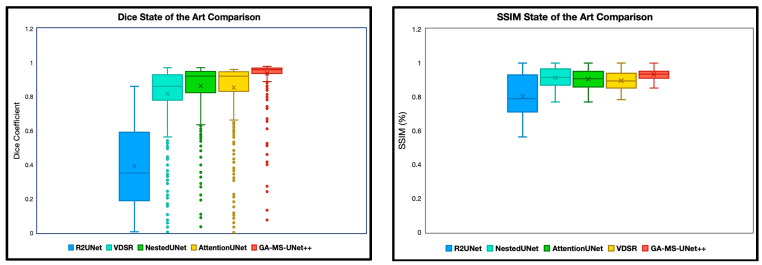
Dice coefficient and SSIM comparison of segmentation performance across five deep learning models on 9.4T MRI scans using manual segmentation as ground truth. Dice scores quantify the spatial overlap between predicted segmentation masks and ground truth annotations, with higher values indicating better segmentation accuracy. SSIM scores were computed between predicted segmentations and ground truth masks, assessing perceptual similarity in terms of luminance. The X notation in between each box and whisker plot represents the mean. GA-MS-UNet++ consistently outperformed the baseline models—including Nested U-Net, Attention U-Net, R2UNet, and VDSR—demonstrating the highest mean Dice score and lowest variance across evaluated slices. These results highlight the model’s robustness in accurately delineating brain structures at ultra-high resolution.

**Figure 3:**
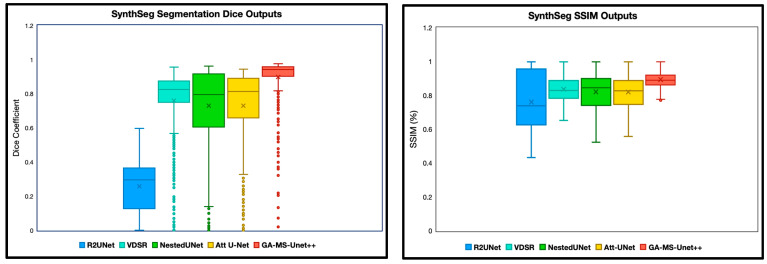
Dice and SSIM comparison across five segmentation models using 9.4T MRI data but with SynthSeg labels as ground truth. Models were evaluated on the remaining scans without manual segmentation ground truth labels. The X notation in between each box and whisker plot represents the mean.

The first experiment we performed was a quantitative analysis using Dice and SSIM to determine the segmentation accuracy across the models. We evaluate our model and baselines using 4 subjects with manual segmentations and 78 subjects with SynthSeg segmentations. To ensure appropriate fairness and relevance, we excluded any slice with a Dice or SSIM value of precisely 0 or 1, as these typically correspond to empty or non-informative slices (common at the beginning or end of MRI volumes). After this filtering, we analyzed the remaining slices for each subject. Dice and SSIM were both included in this study, as Dice captures the overlap, whereas SSIM focuses on the entire image quality. We split the SSIM and DICE results into two categories: manual segmentation ground truth (Figure 2) and SynthSeg segmentation ground truth (Figure 3).

### 3.2. Whole Brain Volumetry

**Figure 4:**
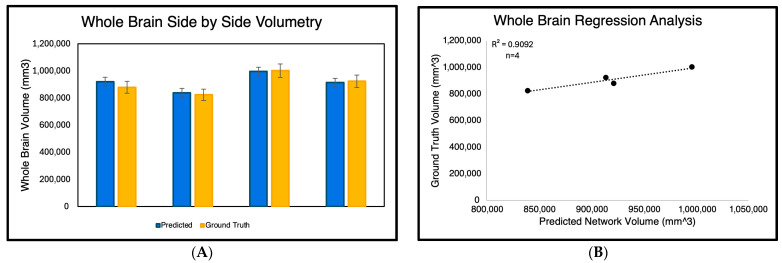
Comparison of predicted and ground truth whole-brain volumes across four manually segmented 9.4T MRI subjects. The left panel (**A**) displays a side-by-side bar graph showing the predicted brain volumes generated by GA-MS-UNet++ and the corresponding manual segmentation ground truth in cubic millimeters (mm^3^) for each subject. The right panel (**B**) presents a regression analysis of predicted versus ground truth volumes, demonstrating a strong positive correlation (R^2^ = 0.9092). These results confirm the volumetric accuracy and reliability of the GA-MS-UNet++ model in high-resolution brain segmentation.

For the volumetry experiment, we evaluated the accuracy of whole-brain volume predictions generated solely by the GA-MS-UNet++ across the four subjects (Figure 4) Each subject’s binarized predicted segmentation output was calculated by counting the nonzero pixels and converting them into cubic millimeters (mm^3^). These predicted volumes were then compared to the ground truth manual segmentations. As shown in the side-by-side bar graph, the GA-MS-UNet++ produced volumes that were close to the ground truth for all four cases. To quantify this relationship further, we performed a regression analysis, yielding a high correlation coefficient of 0.9092, indicating a strong agreement between the predicted and ground truth volumes.

### 3.3. Qualitative Analysis

**Figure 5:**
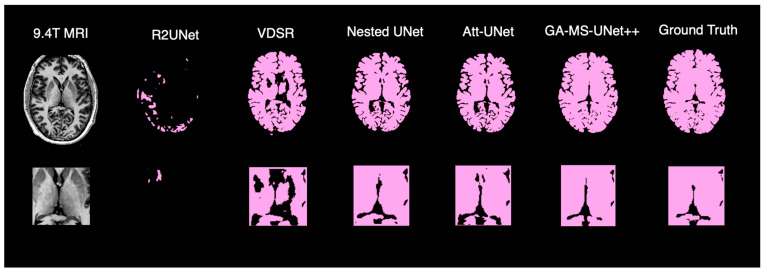
Qualitative comparison of brain segmentation outputs from five different models.

**Figure 6:**
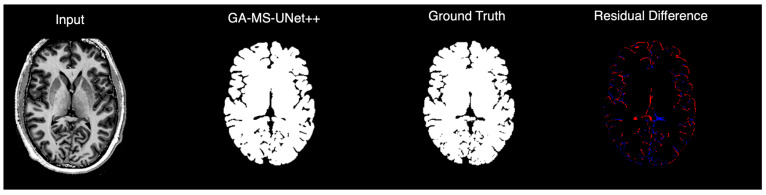
GA-MS-UNet++ segmentation compared to ground truth with residual error map visualization.

To complement the quantitative evaluation, we display the outputs of our model on a random test subject. Figure 5 shows the binarized prediction from all methods for a single brain slice, illustrating that our method yields the most accurate segmentation results. Figure 6 provides a deeper dive into the output of the GA-MS-UNet++, highlighting the residual difference between the ground truth and the output.

### 3.4. Statistical Analysis and Memory Efficiency

We evaluated segmentation performance using accuracy, precision, and recall to provide a more comprehensive assessment among the different error types (Table 1). These metrics were only calculated for the manually segmented images. We conducted a statistical analysis using nonparametric methods. First, a Kruskal–Wallis rank sum test was used to assess the overall differences in Dice scores across all the segmentation models and reference types, making no strict assumptions about the normality of the data. We also used a Wilcoxon signed-rank test for the pairwise comparison between our GA-MS-UNet++ model and each baseline segmentation model, a robust test for matched differences that does not require normal distributions (Table 2). We also reported each model’s inference time, parameter count, memory footprint, and floating-point operations (FLOPs) to benchmark efficiency alongside accuracy (Table 3).

As a benchmark, we compared model outputs and SynthSeg outputs against manual segmentations, confirming SynthSeg as a strong baseline with an overall Dice performance of 0.9192 ± 0.1601, while our model achieved 0.9311 ± 0.1710. Per-subject results showed consistent gains in three of four cases, supporting our hypothesis that our architectural modifications enhance segmentation quality.

To evaluate our model’s generalization to conventional 3T MRI, we tested it on the open-source OASIS dataset [32], where it achieved a Dice score of 0.8651 ± 0.1523 on a set of 446 patients. These findings demonstrate that GA-MS-UNet++ not only performs robustly on ultra-high-field 9.4T data but also transfers effectively to widely used 3T MRI. This cross-field generalization is critical for broader clinical applicability.

## 4. Discussion

In this study, we introduce the GA-MS-UNet++ architecture for brain segmentation at an ultra-high field strength and resolution (9.4T). We conducted thorough statistical testing and rigorous comparisons against state-of-the-art algorithms to ensure our algorithm can be effectively utilized in clinical practice.

We first evaluated our model via Dice scores and SSIM. These evaluations were completed to ensure that the GA-MS-UNet++ performs competitively against other state-of-the-art models in standard quantitative metrics. Our model had a higher Dice score than any of the other four state-of-the-art models and, furthermore, had the smallest standard deviation and spread. The inconsistency of a few of the datapoints was mainly due to slices that were near the edges of the brain. These are challenging to produce accurate segmentations for and are of little use to radiologists.

GA-MS-UNet++ also outperformed the other state-of-the-art algorithms when evaluated via SSIM, albeit with a slightly higher spread. This is expected as SSIM values are generally constrained more. Thus, the other algorithms have a more substantial overlap with the proposed algorithms. SynthSeg segmentation ground truth experiments were performed in addition to the manual segmentation ground truth labels to expand the testing set. Similar results were demonstrated here, with the GA-MS_UNet++ having a higher average and lower spread than the other baselines for both the Dice score and SSIM.

While our model was solely trained on 2D axial slices, we recognize that using partial 3D context could help improve the spatial continuity in segmentation. The 2.5D strategies or overlapping slab-based approaches may help better capture the inter-slice relationships without requiring full 3D volume processing. These methods help preserve anatomical context across the slices and can help enhance the boundary consistency within complex structures. However, the choice to use a 2D design was ultimately motivated by two factors: (1) memory efficiency and faster inference speed for ultra-high-resolution 9.4T data, and (2) clinical emphasis on deployability, where lightweight yet accurate models are favored. Still, future work could compare the GA-MS-UNet++ with 2.5D variants to see whether the spatial continuity gains justify the added computational complexity.

While this study focused on static 2D slices from the 9.4T MRI, the segmentation challenges are even more pronounced in temporally dynamic or multi-modal imaging data. Prior work on multi-modal, vision-based classification and sequence attention modeling suggests that attention-driven architectures like Transformer-based designs are well suited for capturing such dependencies [33]. The gated and residual mechanisms introduced in the GA-MS-UNet++ are thought to be a foundation for extending segmentation frameworks into these more complex domains.

As mentioned previously, brain volumetry calculations are crucial in determining whether an algorithm can be helpful in clinical settings, specifically for diagnosing neurological disorders. In our volumetry experiment, we aimed to compute the whole-brain volume of the ground truth segmentation output and the respective output of the GA-MS-UNet++. Since the algorithm outputs binary segmentation maps, the nonzero voxels were computed in mm^3^. The regression results revealed an R^2^ correlation value of 0.90, demonstrating strong agreement between the true volume and predicted volume for the four subjects with manual segmentations. It is important to note that the volumetric outputs of the study were accounted for when resizing to 256 × 256. Another key limitation of this study is the small number of patients with a manual ground truth. Therefore, we examined individual metrics to identify any outliers. Figure 4 shows each of the four subjects individually with their respective volume outputs. Across all four, there were no significant differences in volume. To ensure the reliability of our Dice score results, we conducted a paired Wilcoxon signed-rank test between our proposed GA-MS-UNet++ model segmentation Dice scores and those from the baseline approaches. The results clearly showed that the GA-MS-UNet++ performed significantly better in all pairwise comparisons, with *p*-values less than 1 × 10^−5^ (Table 2).

In our volumetric validation, GA-MS-UNet++ predictions demonstrated a strong correlation with ground truth brain volumes (R^2^ = 0.90). However, this analysis was based on only four manually segmented subjects and should therefore be interpreted with caution. While the result provides encouraging preliminary evidence that a higher Dice overlap translates into reliable volumetric estimates, it does not, by itself, establish clinical applicability. To mitigate this limitation, we supplemented the analysis with two additional evaluations: (1) large-scale comparison against SynthSeg-derived segmentations across 78 subjects, where GA-MS-UNet++ maintained strong volumetric fidelity, and (2) external testing on the OASIS 3T dataset, which demonstrated generalization with a Dice score of 0.8651 ± 0.1523. Taken together, these complementary findings suggest that the model’s volumetric accuracy is not limited to the small, manually annotated cohort, although larger studies with expert annotations will be necessary to confirm its role in the clinical monitoring of neurodegenerative disease.

Beyond just looking at technical performance numbers, we wanted to understand how better computer accuracy helps in real medical practice. Our volume measurements showed that GA-MS-UNet++ matched human expert measurements very closely (90% correlation), proving that when computers become better at identifying brain structures, the volume calculations become more trustworthy.

Accurate measurements of brain volume are crucial for tracking diseases that affect the brain. In Alzheimer’s disease, certain brain areas like the hippocampus shrink before patients show obvious symptoms, so accurate measurements help with early detection. For multiple sclerosis patients, doctors track both brain lesions and overall brain shrinkage to monitor disease progression and see how well treatments are working.

Upon review, we also observed that the GA-MS-UNet++ demonstrates a slight tendency towards over-segmentation. This behavior explains the higher recall but comparatively lower precision. The model favors including ambiguous boundary regions rather than excluding them. Practically, this results in volumetric outputs that are marginally larger than the ground truth. This conservative bias is likely seen to avoid missing true positives. While this feature may inflate false positives, it also reduces the likelihood of underestimating subtle structures, which can be clinically advantageous in early disease detection.

To test generalizations from the 9.4T to the standard 3T MRI, we evaluated GA-MS-UNet++ on the OASIS dataset. Using SynthSeg to generate reference segmentations, the model achieved a mean Dice score of 0.8651 ± 0.1523, indicating that it can transfer effectively to lower-field data. To confirm that SynthSeg provided reliable pseudo-labels for this purpose, we also compared both SynthSeg outputs and model outputs against a subset of manually segmented cases. SynthSeg achieved a Dice score of 0.9192 ± 0.1601, while our model achieved 0.9311 ± 0.1710, supporting that SynthSeg is a strong baseline and that its labels are appropriate for use as the ground truth in OASIS. Together, these results show that GA-MS-UNet++ performs robustly across different field strengths and that SynthSeg-derived labels offer a practical way to extend evaluation to large external datasets.

Additionally, we applied the Kruskal–Wallis test to assess the differences across all of the models. The test produced an H-Value of 26,281.98, which strongly supports our assertion that models do not perform equally and that the GA-MS-UNet++ stands out in terms of segmentation accuracy. Given that the Dice score is one of the most important indicators of segmentation performance, these statistical results prove the effectiveness of our proposed model and its superiority over other approaches. We also investigated the computational efficiency of our model compared to other state-of-the-art models. We reported a speed of 119.21 m/s, which falls within the range of other algorithms in terms of the inference time to parameter ratio. The accuracy, precision, and recall scores of the model were above 90%, with accuracy and recall being the strongest among all models, and precision performing competitively.

Finally, we perform a qualitative assessment of the binarized segmentation masks from the GA-MS-UNet++ against other state-of-the-art models. Across the axial views, the GA-MS-UNet++ achieves a near-exact match with the ground truth and accurately delineates brain structures while minimizing false positives and false negatives. In contrast, the VDSR and R2UNet exhibit underestimation in key brain regions, such as the frontal lobe. While the Nested U-Net and Attention U-Net offer stronger results, they still exhibit noise and minor inaccuracies across tissue boundaries. When focusing on 9.4T segmentation accuracy, the ventricles and gray–white matter interfaces are preserved with the highest fidelity. The segmented output maintains sharp structural boundaries and captures the fine-scale anatomy and broader brain contours that are characteristic of the 9.4T resolution.

Despite the promising performance of the GA-MS-UNet++ architecture in 9.4T brain MRI segmentation, we recognize that this study has several important limitations. Firstly, the dataset size was limited, with only 12 manually annotated subjects used for training out of the total 86 available scans. This small sample size may compromise the model’s generalizability, particularly when applied to a diverse population. While we tried to incorporate data augmentation, the model’s performance should be validated on larger and more heterogeneous datasets. Secondly, the model was trained on 2D MRI slices rather than on whole 3D volumes. While this reduces the computational demand for both training and inference, it may limit the model’s ability to fully utilize the volumetric spatial context, which is crucial for segmenting complex brain structures. Finally, the dataset consisted of scans from a single 9.4T scanner using imaging parameters. This raises concerns about the model’s adaptability to data from other ultra-high-resolution scanners or even lower-resolution scanners. External validation on these datasets would improve the model’s confidence in clinical applicability. Addressing all three of these limitations in future research will be crucial for translating the GA-MS-UNet++ into reliable clinical tools for ultra-high-resolution brain MRI analysis.

## 5. Conclusions

In this study, we introduce a new algorithm called GA-MS-UNet++, a novel deep learning model specifically designed for high-accuracy brain MRI segmentation on ultra-high-field 9.4T MRI data. Our architecture integrates three main components: (1) multi-scale residual blocks, (2) gated skip connections, and (3) spatial channel attention mechanisms to capture both fine anatomical details and larger structural patterns. The model was ultimately evaluated on metrics such as the Dice score, accuracy, and SSIM, and consistently outperformed the four state-of-the-art segmentation models. Most importantly, the Dice scores were validated through rigorous testing and statistical analyses, including the Wilcoxon signed-rank tests (*p* < 1 × 10^−5^) and a Kruskal–Wallis test (H = 26,281.98), confirming that the GA-MS-UNet++ delivers statistically significant improvements in segmentation accuracy. Qualitative analysis further supports these findings. Compared to the ground truth, GA-MS-UNet++ achieved superior boundary outlining and anatomical resolution, particularly in the ventricles and gray–white matter interfaces. This level of accuracy is crucial, particularly in conditions where small volumetric assessments can indicate neurodegenerative diseases. Volumetric validation also demonstrated a high correlation (R^2^ = 0.90) between the model’s predictions and the ground truth, indicating the potential utility of this approach in monitoring neurodegenerative diseases. Despite there being only a few labeled 9.4T scans, our model performed robustly and maintained consistency across test subjects. Ultimately, as the 9.4T MRI becomes increasingly accessible in research and clinical settings, we hope our model can serve as a foundational tool in enabling precise and automated segmentation. In the future, we even hope our model will have clinical use cases, contributing to improved diagnostic accuracy, streamlined workflows, and better patient outcomes for ultra-high resolution neuroimaging applications.

## Figures and Tables

**Table 1:** Quantitative comparison of segmentation performance across models (manual segmentation output analysis, n = 4). Red-highlighted values represent the highest performing models in each class, respectively.

	R2UNet	VDSR	Nested U-Net	Attention U-Net	GA-MS-UNet++
Accuracy	0.8039	0.9081	0.9434	0.9305	0.9729
Precision	0.8877	0.9721	0.9692	0.9725	0.9002
Recall	0.2175	0.4406	0.6469	0.5525	0.9400

**Table 2:** Wilcoxon signed t-test for paired samples (SynthSeg output dice score analysis, n = 78).

GA-MS-UNet++	*p*-Value
R2UNet	<1 × 10^−5^
VDSR	<1 × 10^−5^
Nested U-Net	<1 × 10^−5^
Attention U-Net	<1 × 10^−5^

Shapiro–Wilk normality test: *p* = 0.0000 (not normal); Kruskal–Wallis test result, H-value: 26,281.98.

**Table 3:** Computational efficiency comparison of segmentation across models (manual segmentation output, n = 4).

	R2UNet	VDSR	Nested U-Net	Attention U-Net	GA-MS-UNet++
Inference (ms)	105.07	93.87	97.07	98.93	119.21
Parameters (M)	39.09	0.66	9.16	34.88	14.66
FLOPs	152.70	43.56	34.62	66.46	20.83
GPU Memory (GB)	0.36	0.07	0.20	0.31	0.24

Abbreviations: floating-point operations (FLOP).

## Data Availability

The UltraCortex dataset used in this study is publicly available at https://openneuro.org/datasets/ds005216/versions/1.0.0 (URL accessed on 1 September 2025).
